# Molecular Characteristics of High-Dose Melphalan Associated Oral Mucositis in Patients with Multiple Myeloma: A Gene Expression Study on Human Mucosa

**DOI:** 10.1371/journal.pone.0169286

**Published:** 2017-01-04

**Authors:** Mette Marcussen, Julie Støve Bødker, Heidi Søgaard Christensen, Preben Johansen, Søren Nielsen, Ilse Christiansen, Olav Jonas Bergmann, Martin Bøgsted, Karen Dybkær, Mogens Vyberg, Hans Erik Johnsen

**Affiliations:** 1 Department of Clinical Medicine, Aalborg University, Sdr. Skovvej 15, Aalborg, Denmark; 2 Clinical Cancer Research Center, Aalborg University Hospital, Sdr. Skovvej 15, Aalborg, Denmark; 3 Department of Haematology, Aalborg University Hospital, Hobrovej 18–22, Aalborg, Denmark; 4 Department of Pathology, Aalborg University Hospital, Ladegaardsgade 3, Denmark; 5 School of Dentistry, Faculty of Health Science, Aarhus University; Vennelyst Boulevard 9, Aarhus, Denmark; University of Catanzaro, ITALY

## Abstract

**Background:**

Toxicity of the oral and gastrointestinal mucosa induced by high-dose melphalan is a clinical challenge with no documented prophylactic interventions or predictive tests. The aim of this study was to describe molecular changes in human oral mucosa and to identify biomarkers correlated with the grade of clinical mucositis.

**Methods and Findings:**

Ten patients with multiple myeloma (MM) were included. For each patient, we acquired three buccal biopsies, one before, one at 2 days, and one at 20 days after high-dose melphalan administration. We also acquired buccal biopsies from 10 healthy individuals that served as controls. We analyzed the biopsies for global gene expression and performed an immunohistochemical analysis to determine *HLA-DRB5* expression. We evaluated associations between clinical mucositis and gene expression profiles. Compared to gene expression levels before and 20 days after therapy, at two days after melphalan treatment, we found gene regulation in the p53 and TNF pathways (*MDM2*, *INPPD5*, *TIGAR*), which favored anti-apoptotic defense, and upregulation of immunoregulatory genes (*TREM2*, *LAMP3*) in mucosal dendritic cells. This upregulation was independent of clinical mucositis. *HLA-DRB1* and *HLA-DRB5* (surface receptors on dendritic cells) were expressed at low levels in all patients with MM, in the subgroup of patients with ulcerative mucositis (UM), and in controls; in contrast, the subgroup with low-grade mucositis (NM) displayed 5–6 fold increases in *HLA-DRB1* and *HLA-DRB5* expression in the first two biopsies, independent of melphalan treatment. Moreover, different splice variants of *HLA-DRB1* were expressed in the UM and NM subgroups.

**Conclusions:**

Our results revealed that, among patients with MM, immunoregulatory genes and genes involved in defense against apoptosis were affected immediately after melphalan administration, independent of the presence of clinical mucositis. Furthermore, our results suggested that the expression levels of *HLA-DRB1* and *HLA-DRB5* may serve as potential predictive biomarkers for mucositis severity.

## Introduction

For three decades, high-dose melphalan, supported with autologous stem cell transplantation (HSCT), has been a component of treatment for patients with newly diagnosed multiple myeloma (MM) [1]. However, melphalan induce adverse effects, including inflammation of the oral and gastrointestinal mucosa (mucositis) and prolonged neutropenia, which necessitates HSCT [2]. Melphalan induced mucositis occurs inconsistently, because although 80% of patients experience some degree of mucositis, only 40% are affected severely [2, 3]. Severe toxicity unfolds as a loss of mucosal integrity, severe diarrhea, and painful oral ulcers; i.e., ulcerative mucositis (UM) [3]. Complicated by bacterial or viral infections, these patients more often experience nausea, diarrhea, febrile episodes, and longer hospital stays compared to patients with mild or no mucositis (NM) [4, 5]. At present, international recommendations consist of infection control and palliative measures for pain relief [6]. Despite intense research efforts, no methods exist for preventing or reducing the duration of mucositis, and no predictive tests are available [7].

The mechanisms of action and metabolism of melphalan are well-described [8]. Melphalan alkylates DNA, which causes cross-links to form between DNA strands, and subsequently, DNA is degraded through apoptosis. The drug is administered intravenously, metabolized in the liver, and excreted through feces and urine. The degree of toxicity depends on renal function, body mass index (BMI), gender, and performance status [2, 9]. However, none of these factors are predictors of UM.

The current model of mucositis pathology is generalized across treatment regimens [10]. Initially, cancer therapy-induced DNA damage activates the intrinsic pro-apoptotic Bax/Bak and p53 pathways, and reactive oxygen species (ROS) are released [11, 12]. Simultaneously, damage to the extracellular matrix induces the release of inflammatory cytokines, which activate the extrinsic apoptotic pathway via tumor necrosis factor alpha (TNF-α) [13, 14]. This release is followed by an inflammatory response, which includes upregulation of the interleukins (IL) IL-1β, IL-6, IL-10, transforming growth factor-beta (TGF-β), nuclear factor-kappaB (NF-κB), and matrix metalloproteinases (MMPs) [15, 16]. This model is mainly based on murine studies and a few human studies, but to the best of our knowledge, no study has focused on patients with MM that were treated with melphalan.

Recent genome-wide association studies (GWAS) of patients that underwent HSCT have implied that UM development is associated with a genetic predisposition, primarily related to immune function [17, 18]. One study included 153 patients with miscellaneous malignancies that underwent HSCT, with the aim of building a predictive network for UM, based on 82 selected single nucleotide polymorphisms (SNPs) [17]. The network was subsequently tested in a cohort of 16 patients, and in the absence of any false positives, the predictive validity of the network was 81.2%. A later study included 972 patients with MM that underwent HSCT, and they identified 11 SNPs located near the matrix metalloproteinase gene that were associated with UM and several known clinical risk factors. The sensitivity of predicting UM was 52% [18]. Apart from the low sensitivity, those studies were limited by their failure to identify phenotypes or causal relationships.

Here, we present a global gene expression study on oral mucosa biopsies and peripheral blood cell samples from consecutive patients with MM that were treated with high-dose melphalan and HSCT. This study aimed to identify new molecular factors that could predict severe oral mucositis.

## Materials and Methods

### Patients

This study included 30 patients, aged 18 years or older, recruited from the Aalborg University Hospital, from September 1^st^ 2010 to September 1^st^ 2012. Patients with MM (n = 20) were recruited from the Department of Hematology. Healthy individuals (CON, n = 10) were recruited for a control group from the Department of Maxillofacial Surgery. Of the 20 MM patients, seven withdrew consent before any intervention; one was missed due to earlier start of treatment, which was not communicated to the research unit; and two withdrew after the first biopsy without giving any reason. The remaining 10 patients provided three sequential buccal biopsies and peripheral blood samples. The first biopsy was obtained immediately before they received high-dose melphalan (day0); the second was obtained after the autologous stem cell reinfusion (day2); and the third was obtained during an outpatient control visit (day21). The CON group comprised 10 healthy, non-smoking, age- and gender-matched individuals. Controls provided one buccal biopsy and peripheral blood sample. One CON individual was later diagnosed with the autoimmune disease, psoriasis (CON09), and hence, this subject was not included in the statistical analysis. The North Denmark Region Committee on Health Research Ethics approved the clinical protocol (N-20100022). Informed written consent was obtained from all patients, in accordance with the Declaration of Helsinki.

All patients with MM underwent a comprehensive, initial evaluation, including a medical history and clinical examination. Age, gender, and Eastern Cooperative Oncology Group (ECOG) performance status were recorded at baseline, in addition to the subtype of MM and the time from diagnosis to entering HSCT. The criteria for determining the level of organ involvement at diagnosis was based on the degrees of elevated calcium, renal failure, anemia, and bone lesions (CRAB criteria) [19]. Furthermore, patients were screened for dental infections and, when indicated, these infections were eradicated prior to chemotherapy.

All patients with MM received a standard induction regimen, which consisted of cyclophosphamide 500 mg/m^2^ delivered intravenously (i.v.) on days 1 and 8; bortezomib 1.3 mg/m^2^ delivered subcutaneously (s.c.) on days 1, 4, 8, and 11; and dexamethasone 20 mg, delivered orally (p.o.) on days 1–2, 4–5, 8–9, and 11–12, repeated every third week, 3 times. After this treatment, patients were primed with cyclophosphamide 2 g/m^2^ and recombinant granulocyte stimulating factor (rhG-CSF), before their circulating CD34^+^ hematopoietic stem cells were harvested with leukapheresis [1]. Only patients without progressive disease were assigned to HSCT. These patients received a high dose of melphalan (200 mg/m^2^), followed by infusion of autologous hematopoietic stem cells. All patients with MM had received standard antiviral, antifungal, and antibacterial treatment, according to department protocols.

### Mucositis and diarrhea assessments

Signs of oral mucositis (OM) were recorded daily for patients with MM during the hospital stay (from administration of chemotherapy to discharge). OM signs were identified according to the WHO oral toxicity assessment worksheet [20], and they included subjective symptoms (pain and ability to eat solid food) and objective findings (erythema, ulceration) in predefined regions of the mouth (lip, check, tongue, floor of the mouth, and soft palate). Grades 0 and 1 (NM) included increasing soreness, with or without erythema, but solid food could be taken. In grades 2 to 4 (UM), food intake gradually declined, due to pain and ulcerations, and parenteral feeding might have become necessary. The maximum OM grade recorded during treatment was considered the patient’s general mucositis experience. Diarrhea was estimated according to the Common Terminology Criteria for Adverse Events (CTCAE), issued by The National Cancer Institute of the National Institutes of Health [21]. In grades 1 to 2, vomiting increases from one to two episodes in 24 h to three to five episodes in 24 h. Grade 3 included more than 6 episodes in 24 h, and grades 4 to 5 were considered life-threatening, and could gradually lead to death. Diarrhea data were gathered retrospectively, from medical records.

### Biopsy

All biopsies were acquired in a standardized manner. First, the mouth was thoroughly rinsed with chlorhexidine and local anesthesia was applied (0.5 ml Citanest®: prilocain 30 mg/ml + felypressin 0.54 μg/ml; Dentsply, York, PA, US). Then, a 5-mm lens-formed biopsy of the buccal mucosa, approximately 1 cm inferior to the papilla parotidea, was taken with a scalpel. The wound was tightly sutured with resorbable vicryl 4.0 (Ethicon, Sommerville, NJ, US). Patients were instructed to rinse twice daily with chlorhexidine until suture removal, after 10 days. One-half of the biopsy was immediately immersed in RNA*later*™ (Ambion, Thermofischer Scientific, Waltham, MA, US) for 24 h; then, it was frozen at -80°C until analysis. The other half of the biopsy was fixed in 10% neutral-buffered formalin, and within 1½ days, it was embedded in paraffin and maintained at room temperature until further preparation.

### Peripheral blood

Within 2 h of taking the biopsy, 15 ml EDTA-mixed venous full blood was drawn. Mononuclear cells (MNCs) were isolated with an in-house standard purification protocol. This protocol follows the manufacturer’s guidelines for Ficoll-Paque^TM^ (GE Healthcare, Little Chalfont, Buckinghamshire, UK); density gradient centrifugation in Leukosep^R^ tubes (Greiner Bio-One GmbH, Frickenhausen, Germany). Purified MNCs were suspended in freezing medium containing 10% dimethyl sulfoxide, in units of 5 million, vital frozen at -196°C in liquid nitrogen, and stored frozen until analysis.

### Gene expression

The frozen oral mucosa samples were homogenized with TRIzol^R^ Reagent (Invitrogen, ThermoFischer Scientific), and total RNA was isolated with the mirVana^TM^ miRNA Isolation Kit (Ambion/Invitrogen, ThermoFischer Scientific) according to the manufacturer’s protocol. RNA amplification was performed with the Ambion^R^ WT Expression Kit (Applied Biosystems, ThermoFischer Scientific), according to the manufacturer’s instructions, starting with 100 ng total RNA, on a TP Basic Thermocycler, real time PCR instrument (Biometra, Göttingen, Germany). The quality of the RNA product was evaluated on the NanoDrop spectrophotometer and the 2100 Bioanalyzer with the Agilent RNA 6000 Nano Kit (Agilent Technologies, Santa Clara, CA, US). We prepared the RNA samples for hybridization to Affymetrix GeneChip Human Exon 1.0 ST Arrays with the Affymetrix GeneChip WT Terminal Labeling and Controls Kit (P/N 901524) (Affymetrix, Santa Clara, CA, US), according to the manufacturer’s instructions. CEL files were generated with Affymetrix GeneChip Command Console Software and deposited at the NCBI Gene Expression Omnibus repository, under number GSE81979. A similar procedure was applied to analyze gene expression in MNCs isolated from blood samples.

### Immunohistochemistry

We cut 4-μm-thick biopsy tissue sections and mounted them on glass coverslips. Following an in-house optimized protocol, tissues were stained with an antibody against the HLA class II Histocompatibility antigen, DR beta 5 chain (HLA-DRB5 center region) with a rabbit polyclonal antibody (no. OAAB06426, Aviva Systems Biology, CA, US). Normal tonsil tissue was used as a positive control. The stained slides were then scanned on a Hamamatsu NanoZoomer slide scanner and analyzed with NDP viewer software. To estimate the number of cells that stained positive for HLA-DRB5, each stained slide was searched for a hot spot; then, this spot was framed with a 0.75×0.4 mm (0.3 mm^2^) rectangle; the area included approximately half lamina epithelialis and half lamina propria. We counted all cells in the frame that were distinctly stained with anti-HLA-DRB5 antibodies. The analyzer was blinded to the mucositis grade.

### Statistical analysis

#### Power estimation of group size

To identify genes that varied more than two-fold between test points with a false discovery rate of less than 0.05% and a power of 90%, we applied the method described by Lee and Whitmore [22], implemented in the R-package, size-power (Qui 2008). We found that 10 patients in each group were sufficient for detecting significant differences.

#### Statistical analysis

All statistical analyses were performed with R [23] version 3.2.0 and Bioconductor packages [24]. The p-values adjusted for false discovery rates were controlled with the Benjamini-Hochberg method [25], for each of the above tests. Adjusted p-values below 0.05 were considered significant.

The CEL files produced by the Affymetrix Expression Console and the probes were preprocessed and summarized to gene level with the RMA algorithm in the Bioconductor package ‘affy’, based on custom CDF files [26]. This preprocessing resulted in the gene expression levels of 38,830 genes for each Exon array each annotated with Ensembl gene (ENSG) identifiers. Patient CON09 was included in the normalizations of the gene expression data, but excluded in the statistical analysis.

With patient ID as a cluster variable, we used the linear model for microarray data (limma package in R), a mixed linear model, and an empirical Bayes approach to test for significant differences in gene expression levels between day2 and day0, and between day21 and day0 [27]. For the peripheral blood samples, we only compared day0 and day21 to baseline, because only two blood samples were analyzable for day2. We performed an unpaired test with the limma package to test for significant differences in gene expression between patients on day0 and controls.

The patients were divided into UM or NM groups, according to their mucositis experience. We used the limma package to detect significant genes that were differentially expressed between the two groups at each time point.

We applied the Mann-Whitney test to test for the relationship between mucositis severity and duration of neutropenia, leukopenia, and thrombocytopenia. We also used the Mann-Whitney test to evaluate differences between groups in the numbers of in-hospital days and years of progression free survival (PFS).

## Results

The clinical characteristics and demographics of the included patients prior to HSCT are shown in **Table 1**. No signs of infection at the site of biopsy were reported. The clinical data collected during the HSCT and at follow up are shown in **Table 2**. UM (grades 2–4) was observed in 4 patients, and NM (grades 0–1) was observed in 6 patients. The average mucositis scores were 1.5 (range 0–4) for the whole cohort, 3.3 for the UM group, and 0.3 for the NM group. The average diarrhea scores were 2.2 (range 1–4) for the whole cohort, 3.3 (range 3–4) for the UM group, and 1.5 (range 1–2) for the NM group. The average hospital stays were 22.6 days (range 16–41) for the whole cohort, 28.8 days (range 21–41) for the UM group, and 18.5 days (range 16–24) for the NM group. The difference in hospital stays was statistically significant (p = 0.020). The duration of neutropenia was not significantly different between UM and NM groups, but thrombocytopenia was significantly prolonged in the UM compared to the NM group (p = 0.047). PFS [28] was not statistically different between the UM and NM groups. The CON and MM groups were comparable in age (CON: age 58 y, range 47–78 vs. MM: age 63.5 y, range 51–69) and gender (CON: females 4/10 vs. MM: females: 6/10).

**Table 1:** Patient characteristics and demographics upon enrollment in the study.

Patient	Age	Gender	ECOG	Weight	MM	CRAB	Induction cycles	Response induction	Standard HSCT	Diagnosis to HSCT
MM01	62	f	2	69	IgG-κ	B	3	No PD	Y	5.3
MM02	51	m	0	110	IgG-λ	B	3	No PD	Y	3.9
MM04	66	f	0	70	IgG-κ	R	3	No PD	Y	3.7
MM05	67	m	1	92	IgG-κ	B	3	No PD	Y	4.6
MM07	67	m	0	63	IgG-κ	C	3	No PD	Y	143
MM09	63	f	1	60	IgG-κ	B	3	No PD	Y	3.9
MM15	69	f	0	72	IgG-κ	A	3	No PD	Y	36.8
MM18	64	f	2	52	IgG-κ	R	3	No PD	Y	5.1
MM19	64	m	0	90	IgG-κ	B	3	No PD	Y	4.1
MM20	62	f	1	97	IgG-λ	B	3	No PD	Y	3.8

Abbreviations: ECOG = Eastern Cooperative Oncology Group performance status at baseline. MM = multiple myeloma subtype. CRAB = end-organ damage at diagnosis (C = hypercalcemia, R = renal failure, A = anemia, B = bone lesions) [19]. Induction cycles = Cyclophosphamide 500 mg/m^2^ i.v. days 1 and 8; Bortezomib 1,3 mg/m^2^ s.c. days 1,4,8, and 11; Dexamethasone 20 mg p.o. days 1–2, 4–5, 8–9, and 11–12, repeated every third week. PD = progressive disease, HSCT = high dose chemotherapy (melphalan 200 mg/m^2^) with autologous stem cell transplantation. Diagnosis to HSCT = months between the diagnosis of MM and the HSCT procedure.

**Table 2:** Patient clinical data during HSCT and at follow up.

Patients	Mucositis grade^1^	Diarrhea grade^2^	Neutro-penia^3^ days	Leuko-penia^4^days	Thrombo-cytopenia^5^ days	In-hospital days	PFS years	Status at follow up^6^
Patients with ulcerative mucositis								
MM04	2	3	4	4	10	41	4.5	CR
MM18	4	4	6	6	12	29	3.1	VGPR
MM19	4	3	10	8	10	21	3.0	VGPR
MM20	3	3	10	10	6	24	3.0	CR
Patients with no/mild mucositis								
MM01	0	2	10	10	14	24	4.1	Relapse
MM02	0	1	12	8	14	17	4.5	CR
MM05	1	2	6	6	14	21	3.2	Relapse
MM07	0	2	8	4	20	16	1.5	Relapse
MM09	0	1	10	8	10	17	1.0	Relapse
MM15	1	1	8	6	10	16	2.2	Relapse

Abbreviations: HSCT = high-dose chemotherapy (melphalan 200 mg/m^2^) and autologous stem cell transplantation; PFS = progression free survival; the surrogate marker for overall survival was defined as the time from entering HSCT to disease progression, death, or follow-up [19]; CR = complete response; VGPR = very good partial response; Relapse = clinical relapse.

^1^ Calculated according to WHO mucositis assessment scale [20]. Patients that experienced mucosal ulcerations during treatment were considered to have ulcerative mucositis, WHO grades 2–4; patients with only soreness or erythema were considered to have none/mild mucositis, WHO grades 0–1.

^2^ Calculated according to the Common Terminology Criteria for Adverse Events [21]

^3^ Neutropenia was defined as <0.5×10^6^/l

^4^ Leukopenia was defined as <0.5×10^9^/l

^5^ Thrombocytopenia was defined as <150×10^9^/l.

^6^According to the International Uniform Response Criteria for multiple myeloma [19].

### Analysis of gene expression in mucosa samples

All 40 biopsies (3×10 patients and 1×10 controls) provided gene expression profiles. No statistically significant differences in gene expression were found between the MM group on day0 and the CON group. Patients with MM showed no significant changes in gene expression between day0 and day21. However, 35 genes in patients with MM showed significantly different expression between day0 and day2 (**Table 3**). The gene expression levels were independent of clinical mucositis. The dominant gene alterations were observed in apoptosis-related genes, followed by genes related to inflammatory/immunologic response, transcription factors, and members of the Histone Cluster family. We also observed alterations in genes related to metabolism.

**Table 3:** Genes altered in the buccal mucosa of patients with multiple myeloma.

Gene symbol	FC		p-value		adjusted p-value	Qualified GO term	Function
Upregulated genes day2 versus baseline							
MDM2	2.69		2.37e-15	3.07e-11		MDM2 oncogene, E3 ubiquitin protein ligase	Apoptosis
EDA2R	2.63		1.85e-15	3.07e-11		Ectodysplasin A2 receptor	Apoptosis
CUL9	2.25		1.26e-15	3.07e-11		Cullin-9	Apoptosis
INPPD5	2.18		8.39e-14	4.66e-10		Inositol Polyphosphate-5-Phosphatase	Apoptosis
TIGAR	2.17		7.08e-10	8.87e-07		Chromosome 1 open reading frame 5	Apoptosis
E2F7	2.06		2.60e-13	1.05e-09		E2F transcription factor 7	Apoptosis
NCR3LG1	2.70		1.14e-10	1.94e-07		Natural killer cell cytotoxicity receptor 3 ligand 1	Immune response
LAMP3	2.26		4.39e-06	0.0011		lysosomal-associated membrane protein 3	Immune response
TREM2	2.12		2.78e-09	2.92e-11		Triggering receptor expressed on myeloid cells	Immune response
FKBP5	2.04		6.66e-05	0.0082		FK506 Binding Protein 5	Immune response
POLH	2.42		1.60e-14	1.24e-10		Polymerase; DNA directed	Transcription
ARNTL	2.40		2.65e-06	0.00080		Aryl hydrocarbon receptor, nuclear translocator-like	Transcription
NFIL3	2.20		4.29e-05	0.0011		Nuclear factor, interleukin 3 regulated	Transcription
ABCA12	4.73		8.87e-07	0.00034		ATP-binding cassette sub-family A, member 12	Metabolism
CEL	4.64		3.68e-15	3,58e-11		Carboxyl ester lipase	Metabolism
CA2	2.57		9.99e-10	1.18e-06		Carbonic anhydrase II	Metabolism
SLC39A6	2.53		1.16e-10	2.00e-07		Solute carrier family 39	Metabolism
SPATA18	2.19		2.16e-12	6.98e-09		Spermatogenesis associated 18	Metabolism
P3H2	2.10		2.28e-09	2.46e-06		Prolyl 3-Hydroxylase 2	Metabolism
F3	2.09		0.00037	0.027		Coagulation Factor III	Metabolism
GLS2	2.01		4.79e-14	3.1e10		Glutaminase 2	Metabolism
WDR63	2.84		7.16e-11	1.35e-07		WD Repeat Domain 63	Unknown
RN7SL519P	2.05		0.00061	0.037		Pseudogene	Unknown
Downregulated genes day2 versus baseline							
SERPINB10		-2.12	1.75e-06	0.00574		Serpin peptidase inhibitor, clade B member 10	Apoptosis
NR1D2		-2.57	9.63e-06	0.00201		Nuclear Receptor Subfamily 1, Group D, Member 2	Transcription
NR1D1		-2.29	0.00015	0.0142		Nuclear Receptor Subfamily 1, Group D, Member 1	Transcription
CIART		-2.38	9.14e-05	0.0103		Circadian associated repressor of transcription	Transcription
HIST1H1A		-2.56	8.31e-07	4.18e-06		Histone Cluster 1, H1a	Transcription
HIST1H1B		-2.04	6.70e-09	6.60e-06		Histone Cluster 1, H1b	Transcription
HIST1H3J		-2.00	8.31e-07	0.00033		Histone Cluster 1, H3j	Transcription
OXGR1		-2.04	8.93e-05	0.010		Oxoglutarate (Alpha-Ketoglutarate) Receptor	Cell signaling
PER3		-2.76	1.77e-05	0.0032		Period Circadian Clock	Metabolism
CYSLTR1		-2.91	8.72e-06	0.0019		Cysteinyl Leukotriene Receptor 1	Cell structure
KIF20A		-2.05	4.65e-08	3.22e-05		Kinesin Family Member 20A	Cell structure
PIK3C2G		-2.06	4.8e-06	0.00121		Phosphatidylinositol-4-phosphate 3-kinase C2 domain-containing gamma polypeptide	Cell growth

Abbreviations: FC = fold change; GO = gene ontology annotation

### Gene expression related to mucositis grade

**Fig 1:**
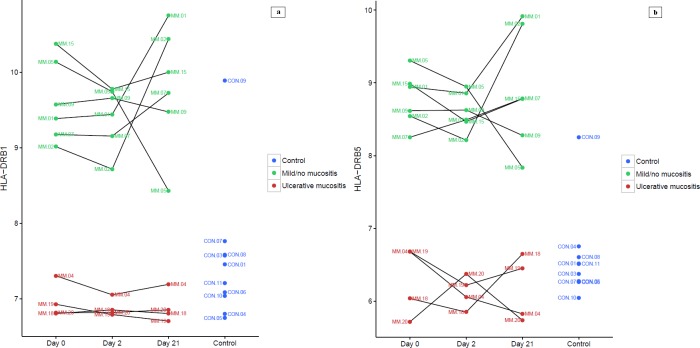
Genes differentially expressed according to mucositis grade. Expression of (a) *HLA-DRB1* and (b) *HLA-DRB5* genes in buccal mucosa biopsies taken at baseline (day0), two days (day2), and 21 days (day21) after high-dose melphalan therapy. Patients with mild/no mucositis (NM) express 6–8 fold more *HLA-DRB1* and 4–5 fold more *HLA-DRB5* than patients with ulcerative mucositis (UM). Melphalan treatment did not affect expression of *HLA-DRB1* or *HLA-DRB5* in either group. One healthy control (CON09) expressed the same high levels of *HLA-DRB1* and *HLA-DRB5* as those observed in the NM group. Subject CON09 was diagnosed with the autoimmune disease, psoriasis. Previous studies have reported that patients with psoriasis were 77% less likely to develop mucositis than patients without psoriasis [54].

When we compared unsupervised gene expression profiles between NM and UM, we found that no genes were significantly differentially expressed in the blood. In contrast, in the biopsies, two genes of the major histocompatibility complex (MHC) Class II: *HLA-DRB1* and *HLA*-DRB5 were significantly differentially expressed at the first two time points (**Table 4**). Patients with UM and CON expressed the same low level of *HLA-DRB1* and *HLA-DRB5*, but patients with NM expressed significantly higher levels (**Fig 1**). The expression levels of *HLA-DRB1* and *HLA-DRB5* were independent of melphalan administration. Of the 10 CON subjects, one patient, CON09, showed high levels of *HLA-DRB1* and *HLA-DRB5* expression, similar to the levels observed in the NM group. We reopened the protocol and returned to the patient to reaffirm his health status. We found that subject CON09 had a mild case of psoriasis that was not reported at the baseline interview. An alternative splicing analysis revealed that patients with NM and the CON09 subject expressed a different isoform of *HLA-DRB1* (NM_001243965) than that expressed by patients with UM (NM_002124.1). However, the difference in *HLA-DRB5* expression between groups was not due to different isoforms.

**Table 4:** Genes altered in the buccal mucosa of patients with multiple myeloma that displayed mild/no mucositis (NM) compared to those that displayed ulcerative mucositis (UM).

Gene symbol	FC	p-value	adjusted p-value	Qualified GO term	Function
NM versus UM day0, before melphalan					
HLA-DRB1	6.27	2.96e-07	0.00573	Human Leukocyte / Major Histocompatibility Antigen Class II DRB1 beta chain	Immune response
HLA-DRB5	5.64	2.7e-07	0.00573	Human Leukocyte / Major Histocompatibility Antigen Class II DRB5 beta chain	Immune response
NM versus UM day2 after melphalan					
HLA-DRB1	5.81	2.01e08	0.00039	Human Leukocyte / Major Histocompatibility Antigen Class II DRB1 beta chain	Immune response
HLA-DRB5	5.56	1.98e09	7.69e05	Human Leukocyte / Major Histocompatibility Antigen Class II DRB5 beta chain	Immune response

FC = fold change; GO = gene ontology annotation.

### Immunohistochemistry

**Fig 2:**
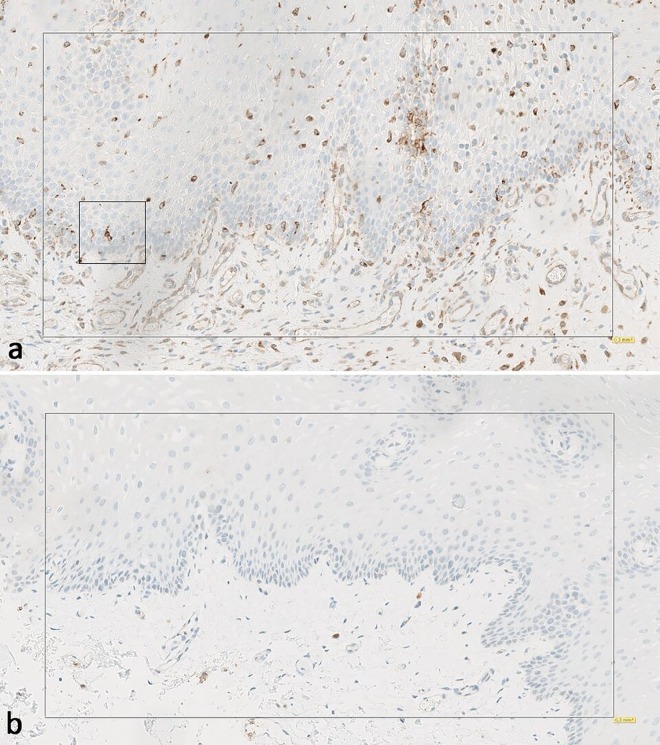
Immunohistochemical analysis of *HLA-DRB5* expression in oral mucosa biopsies. Oral buccal mucosa (×20 magnification) staining shows *HLA-DRB5* expression in the center region (a) High *HLA-DRB5* expression is observed in the patient MM01 with mild mucositis. (b) Low *HLA-DRB5* expression is observed in the patient MM18 with severe mucositis. Generally, when present, cells that stained positive for *HLA-DRB5* are primarily localized in the lower part of the epithelium, near the basal membrane, around the papillae, and in the upper part of the submucosa in close proximity to the basal membrane. A weak, diffuse *HLA-DRB5* staining of the endothelium is also visible. Normal tonsil tissue was included on the slide as a control; these appear identical in (a) and (b). The square insets highlight the morphology of one of the *HLA-DRB5* expressing cells that displayed extensions, similar to those observed in dendritic cells.

**Fig 3:**
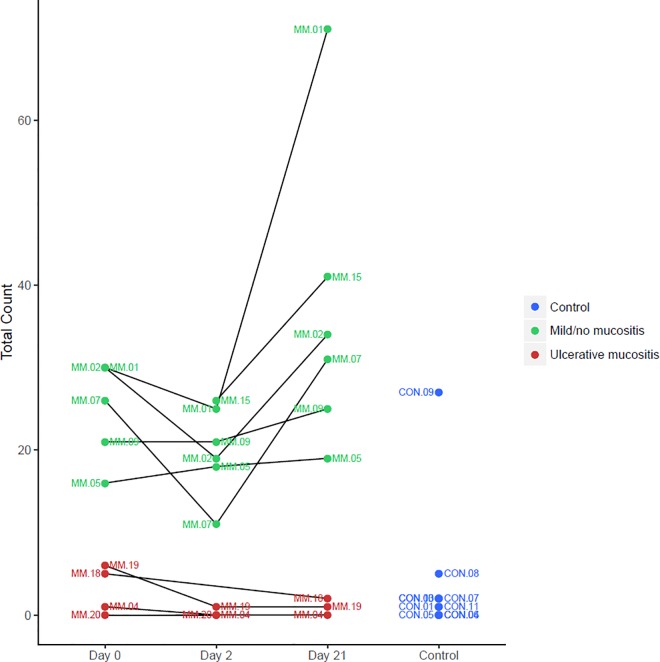
The number of cells that stained positive for *HLA-DRB5* in oral mucosa biopsies. A dotplot shows the cell count performed on oral mucosa biopsies that had been immunostained to detect *HLA-DRB5*. Each biopsy was examined to determine hot spots of expression; then, this section was framed with a rectangle of 0.75×0.4 mm (0.3 mm^2^), and all distinctly stained cells within the rectangle were counted.

In hematoxylin and eosin-stained specimens, no gross anatomical changes were observed in the epithelial or mesenchymal layers. In general, both the epithelium and stroma were represented in equal amounts. However, two specimens that were cut at a tangential angle that revealed only superficial layers were excluded from the histological analysis (MM15_1 and MM18_2). Generally, when present, cells that stained positive for *HLA-DRB5* were localized in the lower part of the epithelial layer, near the basal membrane, around the papillae, and in the upper part of the lamina propria. Faint, diffuse *HLA-DRB5* staining of the endothelium was not included in the assessment. Examples of high *HLA-DRB5* expression/low-grade mucositis (MM01) and low *HLA-DRB5* expression/severe-grade mucositis (MM18) are shown in **Fig 2**. A dotplot of the numbers of cells that stained positively for *HLA-DRB5* is shown in **Fig 3,** and these findings supported the gene expression analysis.

### Gene expression in peripheral blood

Of the blood samples drawn from 10 patients with MM, we successfully performed gene expression profiles in 8 out of 10 drawn on day0 and day21, but only 2 out of 10 drawn on day2 (MM20 and MM05). Consequently, we performed an unsupervised global gene analysis of peripheral MNCs by comparing the MM day0 samples to CON samples (disease vs. healthy) and MM day0 samples versus MM day21 samples (before vs. after treatment). We found that two genes that encoded B-cell surface markers (CD22, CD200) were downregulated in MM day0 samples compared to CON samples, and these genes were further downregulated on day21, though the differences from day0 were not significant. The levels of CD22 and CD200 expression were independent of the mucositis grade.

## Discussion

This study described the gene signature of buccal mucosa samples from patients with MM during HSCT. We found that this signature was dominated by altered expression of inflammatory and anti-apoptotic genes, but expression was independent of the presence of clinical mucositis. Furthermore, we identified a specific isoform of the immunomodulatory gene, *HLA-DRB1*, which may serve as a biomarker for mucositis severity.

The process that leads to mucositis is triggered immediately upon initiation of cancer therapy and before any visible macroscopic damage [10]. Moreover, eventual UM coincides with neutropenia, within 7–10 days of starting chemotherapy. Therefore, to avoid compromising patients with neutropenia, we acquired the second biopsy before the onset of neutropenia. With this approach, the biopsy was unlikely to comprise disintegrated tissue that, presumably, would be dominated by inflammatory mediators. Instead, we aimed to gain insight on the cellular processes that gave rise to the inflammatory state. Mucositis lasts for approximately 7 days, and then, it spontaneously resolves. We acquired the third biopsy on day 21, when the mucosa was fully restored.

Little is known about the effects of melphalan on normal epithelium, but in cancer cells, melphalan induces oxidative stress and upregulates a wide range of apoptosis-related genes [8, 29], consistent with our findings of *EDA2R* upregulation. *EDA2R* encodes a TNF receptor that mediates the activation of NF-κB and jun-N-terminal (JNK) pathways, which lead to caspase-initiated apoptosis. Previous studies reported that these pathways were activated in buccal mucosa of patients with various cancers that received HSCT [30–32], and in gastrointestinal mucosa of patients treated with 5-fluoruracil [33]. In contrast, we found upregulated expression of *INPP5D*, which encodes a membrane protein in hematopoietic cells. The INPP5D protein negatively regulates JNK signaling and limits Fas-FasL-induced apoptosis in T-cells found at mucosal surfaces [34]. In addition, we identified five genes involved in suppressing the pivotal p53 apoptotic pathway. Four of these were upregulated: *MDM2*, *CUL9*, *E2F7*, and *TIGAR*; and one was downregulated: *SERPINB10*. The *MDM2* gene encodes a protein ligase that ubiquitinates p53, and thus, inhibits p53-mediated cell cycle arrest and apoptosis. A previous gene expression study on three patients with acute myeloid leukemia used the same time intervals between biopsies that we used, and they found *MDM2* upregulation [35]. In an array of studies, p53 has been identified as a key regulator of apoptosis, which leads to mucositis [11, 12, 36]. However, the gene alterations associated with apoptosis observed in our study, including *EDA2R*, did not depend on the level of clinical mucositis.

We found several genes related to transcription that were altered to favor DNA repair. For example, *POLH* was upregulated; *POLH* encodes a specialized DNA polymerase that accurately replicates UV-damaged DNA. Conversely, members of the histone cluster family (e.g., *HIST1H1A*), *NR1D1*, and *NR1D2* were downregulated. These results implied that defense against apoptosis and DNA damage was a central objective in the initial stage of mucositis. Importantly, this objective was independent of the mucositis grade, which implies that other factors must be involved in distinguishing UM and NM.

The immune response was activated at an early stage, through the upregulation of *TREM2* and *LAMP3* on day2. Both these genes encode DC membrane proteins that contribute to T-cell activation and mucosal inflammation. The *LAMP3* gene is specifically expressed in mature DCs [37, 38]. TREM2, which is expressed on both DCs and macrophages, can bind and phagocytose yeasts, Gram positive bacteria, and Gram negative bacteria [39], which are commonly present in the oral cavity [40]. Generally, DCs are potent antigen-presenting cells that respond to microbial exposure by secreting abundant cytokines; e.g., IL-12 and type I interferon. In turn, IL-12 mobilizes natural killer (NK) cells. The *NCR3LG1* gene, which encodes a ligand that triggers NK cells, was also upregulated [41]. Several studies have shown that an important aspect of mucositis pathology is the thinning of the epithelium, in combination with changes in the composition and concentration of the oral and gastrointestinal microbiota [13, 42, 43]. Our results confirmed the notion that the host immune response towards the microbiome played a dominant role, early in mucositis pathogenesis; however, these responses were not associated with mucositis severity.

Among several genes associated with metabolism, *ABCA12* and *CEL* were upregulated 4.7-fold and 4.6-fold, respectively, on day2. *ABCA12* encodes a membrane transporter protein primarily involved in the keratinocyte lipid-barrier that maintains homeostasis in the epidermis [44]. To the best of our knowledge, no previous study has described a role for *ABCA12* in the mucosa, but it most likely performs a similar function of barrier protection. *CEL* encodes a lipase with multiple functions in lipid metabolism; it is also expressed in macrophages [45]. The expression levels of both these genes were unaffected by clinical mucositis.

### Gene alterations associated with clinical mucositis grade

When we compared the gene expression profiles between the six patients with NM and the four patients with UM, we found two genes that were more highly expressed in NM patients: *HLA-DRB1* and *HLA-DRB5* (**Fig 1**). The *HLA-DRB1* and *HLA-DRB5* genes are related members of the MHC Class II family, located on chromosome 6p21.32. They encode surface proteins that are almost exclusively expressed on specialized antigen presenting cells, including macrophages, B-cells, and DCs or Langerhans cells [46, 47]. These surface receptors function as a ligand for the T-cell receptor, and their primary function is to capture potentially foreign antigens on the cell surface and to present them for recognition by CD4+ T-cells [48]. Thus, they form a communication between the innate and adaptive immune systems, and determine whether to bring forth resistance or tolerance, in addition to taking up and processing dying cells [49].

Several pharmacogenomic GWAS studies were recently performed on drug toxicity, which showed that HLA Class I and II paralogs were associated with toxicity [50] or inflammatory mucosal conditions [51, 52]. Even more interestingly, certain HLA-DRB1 alleles (HLA-DRB1*15) have been detected in patients with MM that were exposed to bisphosphonates and developed osteonecrosis of the jaw [53].

We conducted a search for alternatively spliced variants of *HLA-DRB1* and *HLA-DRB5* and found two isoforms of *HLA-DRB1* (NM_001243965 and NM_002124). According to the UCSC genome browser, NM_001243965 harbors six exons, and NM_002124 harbors an extended isoform within seven exons. We found that patients with NM expressed the longer transcript variant of *HLA-DRB1* (NM_002124), and patients with UM and healthy subjects (CON) expressed the shorter variant (NM_001243965). No splice variant was found for *HLA-DRB5*; however, that gene was expressed at different levels. We found that *HLA-DRB5* was expressed 4.5 to 5 times more frequently in the NM group than in the UM group, in the first two of three biopsies. These findings were confirmed in an immunohistochemical analysis of HLA-DRB5 in the biopsies (**Fig 3**). Furthermore, cells that stained positively for HLA-DRB5 were localized primarily in the epithelium and submucosa, relatively close to the basal membrane, and these cells displayed a morphology similar to DCs (Langerhans cells).

In contrast to the other healthy subjects, CON09 (a patient with psoriasis) expressed the long transcript variant of *HLA-DRB1* and a high level of HLA-DRB5 protein, similar to patients in the NM group. Previous reports have indicated that patients with psoriasis are 77% less likely to develop mucositis [10, 54]. Psoriasis is an auto-inflammatory skin disorder with reduced apoptosis. It is known that patients with psoriasis express certain *HLA-DRB1* alleles [55]. Our results suggested that mechanisms related to inflammatory and/or apoptotic pathways may be common in psoriasis and low-grade mucositis in MM. In addition, *ABCA12* expression was upregulated in CON09 compared to the other healthy subjects. Previous gene expression studies on patients with psoriasis confirmed this finding [56].

The two recent GWAS studies on patients that received HSCT identified SNPs near the locus of MMP and other genes related to inflammation, but none related to *HLA-DR* [17, 18]. Other recent studies found a major role for MMPs in mucositis pathology [57]. We did not find any changes of that nature in our material. The phenotypes described in our study may provide additional information to guide future GWAS studies [50]. Recent studies have shown that induction therapy with immune modulating agents reduced the frequency and severity of mucositis [58]; our results may provide additional knowledge to elucidate the development of those therapies.

In peripheral blood, we did not find any differences in gene expression between NM and UM groups, at any time point. However, among all patients with MM, *CD22* and *CD200* were downregulated on day0 compared to controls. Both these genes encode cell membrane glycoproteins of the immunoglobulin superfamily. *CD200* is expressed in multiple cell types, including B-cells, T-cell subsets, DCs and endothelial cells. In contrast, *CD22* is exclusively expressed on mature B-cell lineages [59, 60]. Low CD200 expression has been linked to prolonged survival among patients with MM [61]. In our cohort, the lowest CD200 expression levels were observed among patients with UM; however, the levels were not significantly different between UM and NM groups.

### Study strengths and limitations

There was some concern that breaking the mucosal barrier by taking a biopsy during chemotherapy might lead to potential fatal infections. We could reject this concern, because none of our patients experienced any infection related to the biopsy; only mild discomfort was reported. This finding was also reported in previous studies [15, 35, 36, 62]. Therefore, we concluded that our method would be safe for patients undergoing HSCT, provided that the second biopsy is taken before the onset of neutropenia. The major limitation of the study was the low number of subjects. We designed the study to identify genes that were altered by more than 2-fold between time points, with a false discovery rate of less than 0.05% and a power of 90%. However, because our method of harvesting human mucosa during high-dose melphalan treatment was controversial, we sought to include the least possible number of patients required to draw valid conclusions. However, we recognize that the power of this study was set to estimate any fold-changes above two, and false negative findings may be concealed. Consequently, we did not expect to elucidate the full, true picture; nevertheless, we brought to light some important biological associations, which have motivated us to continue this research and confirm the results in a larger cohort.

## Conclusions

Currently, there is a great need to develop a clinically applicable method for identifying potential susceptibility to toxicity among patients before treatment initiation. We found that patients with NM displayed upregulations of *HLA-DR1* and *HLA-DRB5* compared to patients with UM and healthy individuals. These genes encode proteins expressed on the surface of antigen presenting cells in mucosa, which suggested that the immune response might play a major role as a primary effector in UM. Indeed, the results suggested that expression of a certain isoform of *HLA-DRB1* might diminish the inflammatory response to melphalan toxicity. However, because the levels of *HLA-DRB1* and *HLA-DRB5* expression were constant throughout treatment, this isoform may serve as a predictor of UM. The findings in this study were based on a small number of samples, and thus, our results must be validated in a larger patient cohort.
